# Unusual presentation of a splenic infarction

**DOI:** 10.1016/j.radcr.2024.08.124

**Published:** 2024-09-13

**Authors:** Kolos K. Nagy, Caroline J. Cushman, Andrew F. Ibrahim, Seshadri D. Thirumala, James Montgomery

**Affiliations:** a School of Medicine, Texas Tech University Health Sciences Center, Lubbock, TX, USA; b Department of Pathology, Covenant Medical Center, Lubbock, TX, USA; c Department of Interventional Radiology, Covenant Medical Center, Lubbock, TX, USA

**Keywords:** Splenic infarction, Splenic neoplasm, Splenic lesion, Splenectomy, Case report

## Abstract

A 41-year-old male with a history of tobacco and alcohol use presented to our clinic for a follow up of an incidentally diagnosed splenic mass. The patient was sent for further diagnostic evaluation, and computed tomography showed a large splenic mass with heterogenous enhancement raising concern for neoplasm. Due to the uncertain nature of the splenic lesion and high complication rate of percutaneous splenic biopsy, a splenectomy was performed. The specimen was sent to pathology, and the report favored neoplasm but was inconclusive. The samples were sent to another institution for a consult, where the patient's spleen was determined to be the result of a previously suffered hemorrhagic infarct. This case demonstrates the difficulty of diagnosing splenic lesions using diagnostic imaging and the discrepancy that may occur between radiology and pathology assessments. In the setting of uncertain pathology, the removal of what might be a functional spleen is often preferred over a percutaneous biopsy due to concerns of intraabdominal bleeding and tumor dissemination.

## Introduction

The spleen plays a critical role in immunological defense, being the largest lymphoid organ in the body [1]. There is considerable literature discussing the differential diagnosis of splenic lesions. However, diagnosis and management may be difficult due to their rarity, lack of clinical signs in their setting, and their diagnosis being primarily dependent on imaging characteristics [2].

Splenic infarcts are rare, and often go undiagnosed until an incidental encounter which may be related to complaints of persistent abdominal pain, nausea, vomiting, and constipation. A splenic infarction occurs due to tissue necrosis from the interruption of arterial blood supply to the spleen and subsequent parenchymal ischemia. Ultrasound (US) and computed tomography (CT) imaging may be used to diagnose splenic infarcts in patients with nonspecific abdominal pain. Appearance varies, but an infarction may present as a hypoechoic (US) or hypodense (CT) lesion with irregular contours, which may be segmental or involving the whole spleen. Hematological disorders, systemic thromboembolism, cardiovascular disorders, autoimmune diseases, trauma, intraabdominal surgery, and infection are all risk factors for splenic infarcts [3]. A hematoma is a localized collection of blood outside of blood vessels. A splenic hematoma may be a complication of blunt force abdominal trauma, infection, malignancy, hematological disease, or in rare cases, they may have no attributed causative factor [4].

Splenic tumors are rare and are often found incidentally. Splenic tumors can be benign or malignant primary tumors, or can be a result of metastasis from gastric, colon, or ovarian cancers [5]. Splenic involvement of B-cell lymphomas is well described in literature while the involvement of peripheral T-cell and natural killer cell neoplasms is limited, leading to a degree of uncertainty in some cases [6]. Literature has raised concerns over the limitation of diagnostic imaging in splenic lesions, and without conclusive history, physical examination, and laboratory results, a laparoscopic splenectomy is chosen as the diagnostic and curative approach without attempt to salvage viable tissue due to concern that a percutaneous biopsy may result in intraabdominal bleeding and tumor dissemination [5,7]. Here we present a case of an incidentally found splenic mass that initially raised concern for and favored neoplasm but had inconclusive radiology and pathology assessments. After consultation and further review, the splenic mass was determined to be the result of a hemorrhagic infarction of uncertain timeline and etiology.

## Case review

A 41-year-old male presented to our clinic for a follow up of a recently diagnosed splenic mass. Two months before the visit, the patient fell and had persistent abdominal pain, and a week after unresolving symptoms, he presented to the radiology department for imaging where he was diagnosed with a splenic mass.

In the follow up visit, the patient stated that general pain had been present for years. Patient reported nausea, vomiting, and weight loss for years, but had seen no stool changes. He stated that pain increased with activity. The patient reported significant unexpected weight loss, most of which happened 8-9 months prior to diagnosis of the splenic mass. He reported that vomiting increased lying down or eating hard foods. He reported no activity change, appetite change, chills, diaphoresis, fatigue, or fever.

The past medical history of the patient included arthritis, back pain, groin injury with surgery, and a motor vehicle accident nearly 30 years earlier with rib fractures that required a chest tube and surgery. Patient reported smoking 2-3 packs of cigarettes a day and had a 45.00 pack-year smoking history. He reported alcohol use of about 7.0 standard drinks of alcohol per week. The patient stated that he did not use drugs at the time. Family history included fatal metastatic colon cancer in father. On physical exam, patient had a BP of 149/87, pulse of 60, temperature of 97.3, respiration rate of 16, SpO2 94%, and a BMI of 30.27 kg/m^2. The patient's comprehensive metabolic panel showed elevated Chloride at 111 mmol/L (98 – 109 mmol/L) and elevated ALT at 65 U/L (16 – 61 U/L). Complete blood count showed elevated RDW-CV at 15.9% (11.5%-15.3%). The patient had a CEA of 1.8 (0.0-3.0 ng/mL), lactate dehydrogenase of 125 (87-241 U/L), and normal ECG 12 lead.

**Fig. 1:**
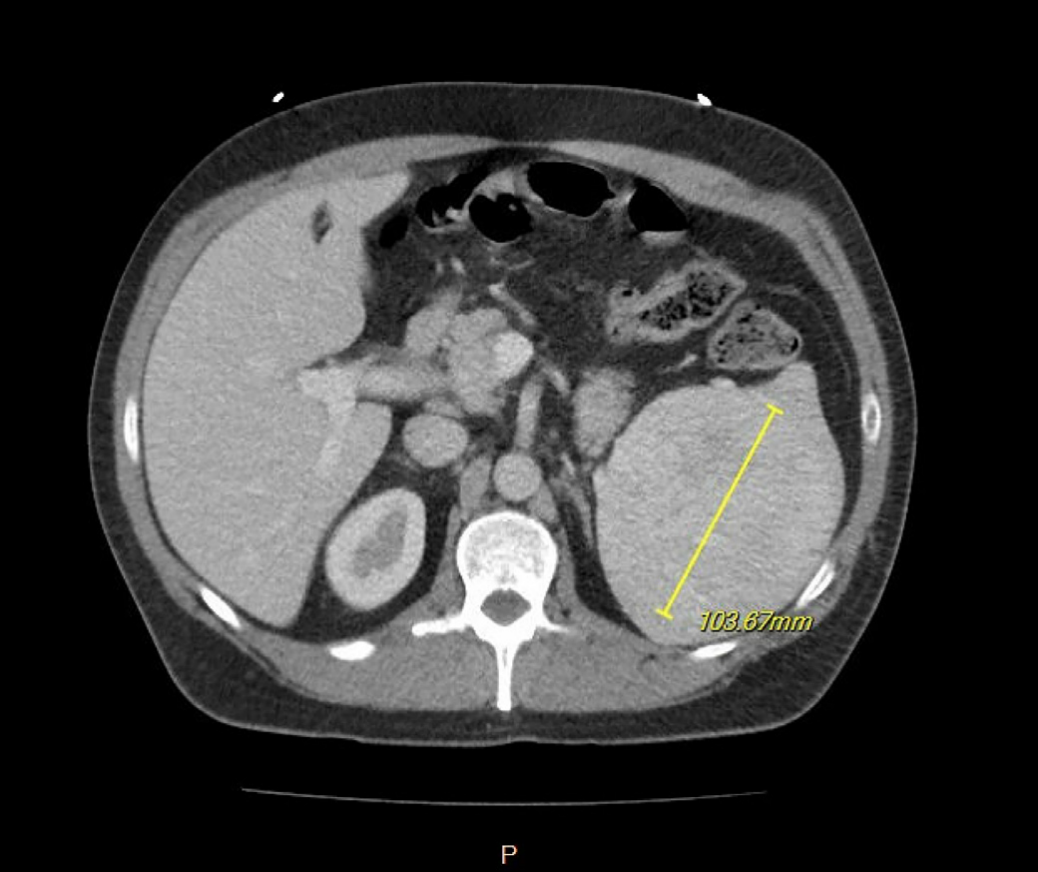
Axial view CT w/ contrast of splenic mass.

**Fig. 2:**
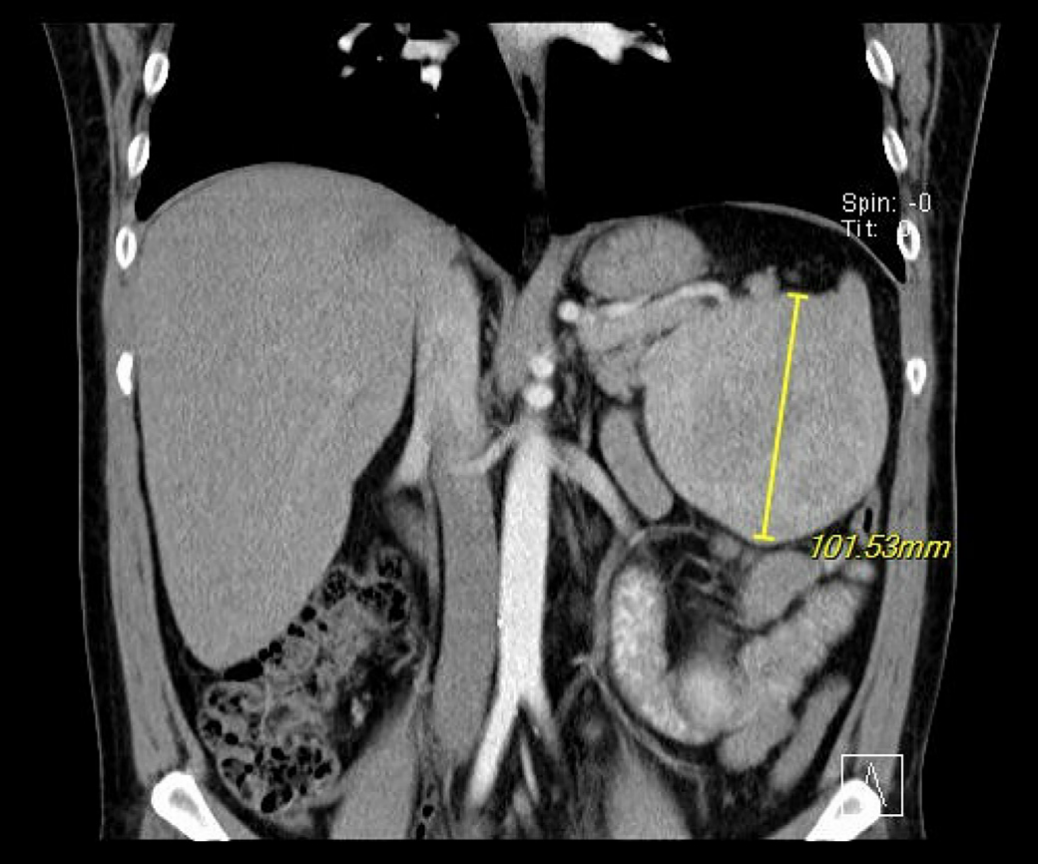
Coronal view CT w/ contrast of splenic mass.

CT of abdomen and pelvis with contrast showed a large splenic mass with heterogenous enhancement measuring up to 9.5 × 10.3 × 11.7 cm in maximal transverse by AP by craniocaudal dimensions with no calcifications, unchanged from the initial visit (Fig. 1, Fig. 2). The remainder of the spleen was unremarkable, Contrast CT showed Avg. 132.9 HU (40-60 HU) enhancement of the splenic lesion. No free fluid was seen in the abdomen, bowel loops were not dilated, there was no colonic wall thickening, appendix appeared normal, no significant lymphadenopathy was present, visualized regional osseous structures appeared unremarkable, and visualized loops of bowel were unremarkable. The splenic vein of the patient was markedly dilated.

The size, morphology, and enhancement of the splenic mass raised concern of neoplasm, but hematoma was also a possibility given that the patient had prior trauma by falling. A percutaneous biopsy was avoided due to risk of missed diagnosis and bleeding, and the patient was recommended for a laparoscopic splenectomy. Patient was informed of the risks of surgery, received all required vaccines, and proceeded with the splenectomy less than a month later. No complications were reported from the splenectomy and patient was discharged less than 48 hours later.

**Fig. 3:**
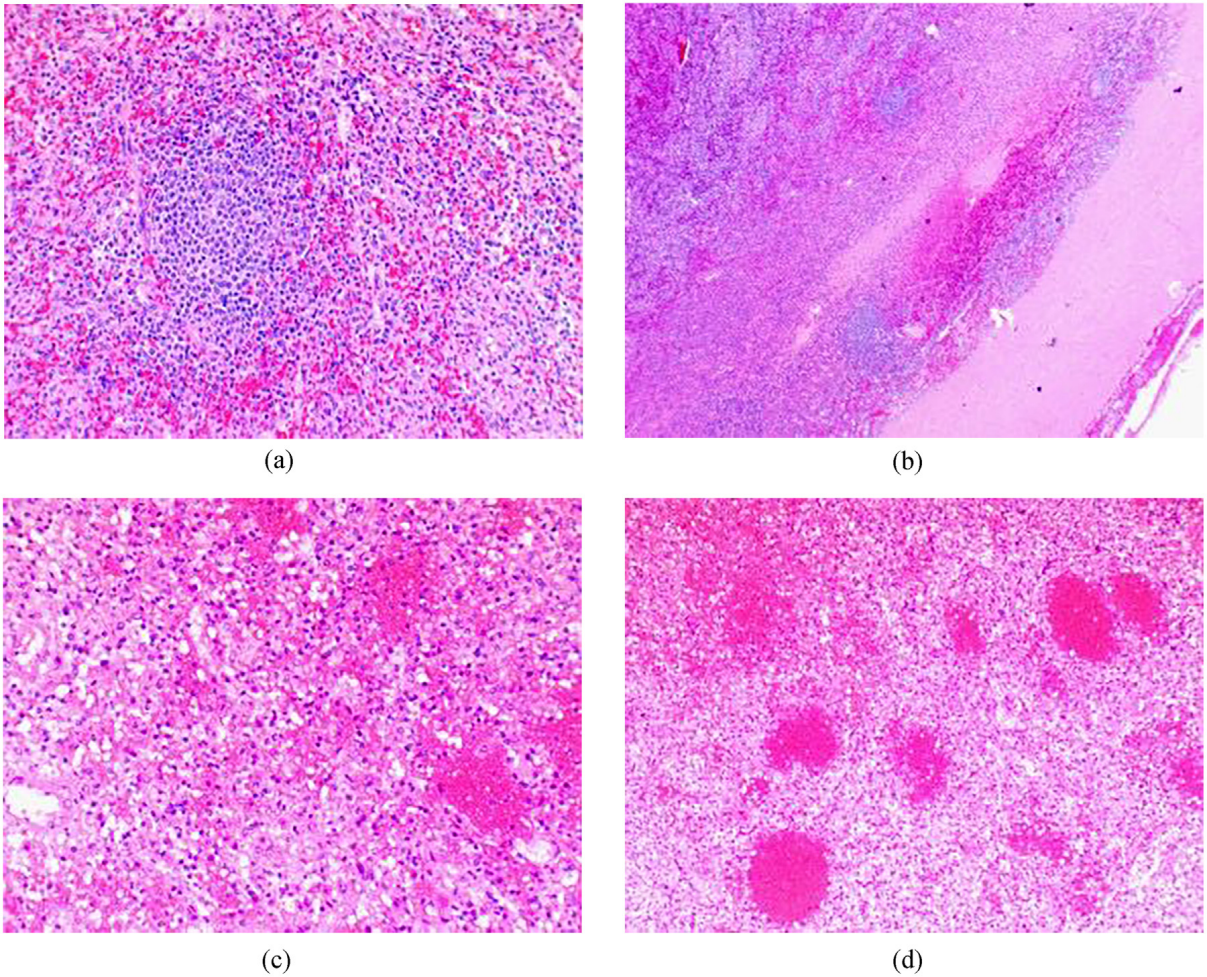
(A) Low power (100x) view of normal spleen parenchyma. (B) Low power (200x) view of subcapsular area showing infarction. (C) Intermediate (200x) view of infarction. (D) High power (400x) view showing infarction.

The specimen was sent to surgical pathology. The splenic red pulp was expanded and contained a mixture of small and large atypical lymphoid cells. T-cell markers CD3, CD5, and CD10 stained on some but not all large cells. CD20 was negative in majority of cells. BCL2 stained many cells in the red pulp but was negative in germinal centers of lymphoid follicles in the white pulp. BCL6 showed nuclear staining in small and large, atypical cells. CD15 was positive in cells of myeloid origin, and CD30 stained activated cells. Cyclin D1 was negative, and CD138 showed no significant increase in number of plasma cells or plasmocytic lymphocytes. Antibodies to kappa and lambda light chains showed the plasma cells to be polyclonal. CD17 was negative. Tissue was noted for atypical large cell infiltrates suspicious for peripheral T-cell lymphoma (Figs. 3A-D). Flow cytometry revealed no diagnostic abnormalities. The specimen was then sent to another institution for a consult and genomic testing.

The consult identified a large spleen measuring 13.5 × 9.2 × 7.0 cm in dimension and 551 grams in mass, described as having a dark red surface without solid masses or gross lesions. Histologic sections showed areas of large red pulp composed of dilated vascular channels with extensive hemorrhage, congestion, and necrosis consistent with hemorrhagic infarct. In an adjacent area of the spleen, an organizing thrombus involving a large blood vessel was identified. In the uninvolved spleen, few megakaryocytes were present in the red pulp. The area consistent with infraction showed destroyed red pulp as shown by the CD8 and ERG antibodies, and CD30 was negative. There was no morphologic evidence of malignant neoplasm, and the specimen was diagnosed as benign likely being the result of a previously suffered hemorrhagic infarction.

## Discussion

A normal spleen appears uniformly homogenous on noncontrast CT, inhomogeneous in the early, and uniformly enhanced on venous phases. Splenomegaly is not exclusive to hematological malignancies, as 30% of tumor infiltrated spleens are of normal size, and splenomegaly can occur in the absence of tumor involvement. Calcification of splenic lesions is rare, independent of etiology [1].

Past trauma or infarction normally presents as a linear or triangular shape, either in solitary or multiple masses but they may be variable in appearance depending on extent and age of the lesion, which may make them difficult to discern on diagnostic imaging. An incidental finding of a previously acquired infarction or traumatic lesion may be confounding on diagnostic imaging and raise concern for a malignant or novel condition [8].

An infarction can compromise the integrity of blood vessels in the spleen because of tissue damage and the inflammatory response, resulting in hemorrhage and subsequent hematoma [3]. The continuous border, enhancement, and large, spherical shape of the mass in this patient's spleen made the definitive diagnosis of an infarction difficult with diagnostic imaging, and primary neoplasm in the differential could not be definitively ruled out.

Splenic lesions can be divided into cystic and solid lesion. Both cystic and solid lesions can be further subdivided based on solitary or multiple masses. Cystic lesions with solitary masses include epithelial cysts, pseudocysts, and echinococcal cysts, and cystic lesions with multiple masses can be divided into infectious diseases and lymphangiomas. Solid lesions with solitary masses can be divided into vascular and nonvascular lesions. Solid solitary vascular lesions include hemangiomas, hamartomas, sclerosing angiomatoid nodular transformations (SANT), and angiosarcomas, and solid solitary nonvascular lesions include lymphomas and inflammatory pseudo tumors. Solid lesions with multiple masses include inflammatory conditions, littoral cell angiomas, and metastasis [2]. Littoral cell angioma was heavily considered in the differential diagnosis of both radiology and pathology in this patient. Littoral cell angioma may present as a singular dominant mass or one with multiple masses. Lesions of the spleen are rare, and primary neoplasms are even more uncommon and present a diagnostic challenge [5].

**Fig. 4:**
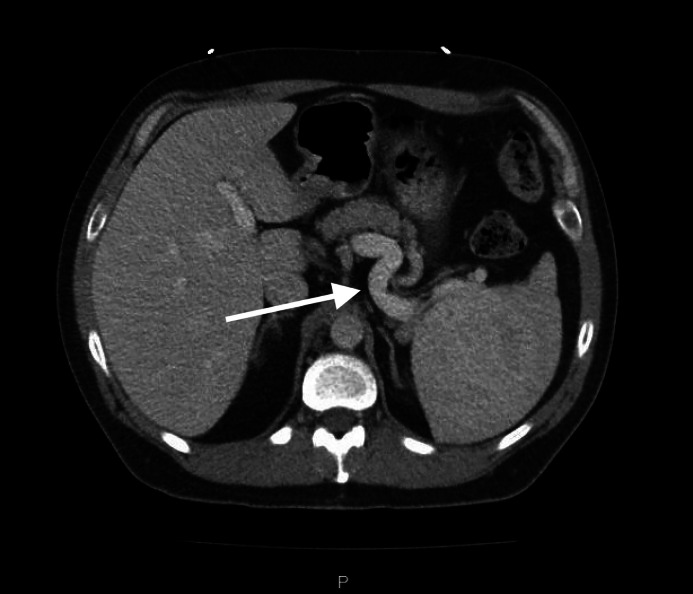
Axial view CT w/ contrast demonstrating enlarged splenic vein.

The mechanism by which this patient developed the lesion remains unknown. An undiagnosed splenic infarct, possibly the result of trauma and/or compromised vascular integrity from a history of tobacco and alcohol consumption, may have resulted in rupture and leakage of blood into the spleen with subsequent hematoma [3]. Hematomas rarely show enhancement because they form in a single location, and not only did the CT reveal enhancement of the lesion, but this patient's hematoma infiltrated through the lymphatic tissue of the red and white pulp. The enhancement of this patient's lesion would not be traditionally consistent with hematoma, and the dilated splenic vein can be interpreted as evidence of a growing tumor with increased demand for blood supply (Fig. 4) [8].

## Conclusion

The etiology of splenic lesions can vary widely. Determining the cause and timeline of such lesions can be challenging, particularly in cases where patients are asymptomatic, lack a clear inciting event, and have inconclusive diagnostic or laboratory results, as was the case in this patient. Physicians should maintain a high index of suspicion for splenic pathology, even in asymptomatic patients, and consider further diagnostic studies to elucidate the underlying etiology and further guide management strategies.

## Patient consent

Consent for the publication of this case report was obtained from the patient.
